# Deterministic controlled enhancement of local quantum coherence

**DOI:** 10.1038/s41598-022-26450-1

**Published:** 2022-12-27

**Authors:** Nikola Horová, Robert Stárek, Michal Mičuda, Michal Kolář, Jaromír Fiurášek, Radim Filip

**Affiliations:** Department of Optics, Faculty of Science, Palacký University, 17. listopadu 1192/12, 779 00 Olomouc, Czech Republic

**Keywords:** Optics and photonics, Information theory and computation, Quantum physics

## Abstract

We investigate assisted enhancement of quantum coherence in a bipartite setting with control and target systems, which converts the coherence of the control qubit into the enhanced coherence of the target qubit. We assume that only incoherent operations and measurements can be applied locally and classical information can be exchanged. In addition, the two subsystems are also coupled by a fixed Hamiltonian whose interaction strength can be controlled. This coupling does not generate any local coherence from incoherent input states. We show that in this setting a measurement and feed-forward based protocol can deterministically enhance the coherence of the target system while fully preserving its purity. The protocol can be iterated and several copies of the control state can be consumed to drive the target system arbitrarily close to a maximally coherent state. We experimentally demonstrate this protocol with a photonic setup and observe the enhancement of coherence for up to five iterations of the protocol.

## Introduction

The principle of superposition is a fundamental property of quantum systems. This principle gives rise to quantum coherence, that has been identified in recent years as an important resource in various areas of quantum information sciences, quantum technologies and quantum thermodynamics. A rigorous resource theory of quantum coherence has been developed^1,2^ yielding quantification of quantum coherence of both pure and mixed states, and several classes of incoherent quantum operations, i.e. operations that cannot generate quantum coherence from input incoherent states, have been identified. The concept of quantum coherence is tightly connected to the observation that some states of quantum systems can be more easily prepared than others. In particular, one identifies a specific basis of incoherent states and it is postulated that these free states possess zero coherence. Within this framework of quantum resource theory, operations that can generate or increase quantum coherence are considered to be more costly than the free operations that do not increase the coherence.

Manipulation with quantum coherence has been the subject of numerous recent theoretical^3–14^ and experimental^15–21^ studies. Of particular interest is the relationship between quantum coherence and quantum entanglement, that also represents a fundamental resource, related to but distinct from quantum coherence. This relationship becomes particularly relevant in the assisted distillation of quantum coherence^4,10,15,18^. Here one considers a bipartite quantum system consisting of target subsystem A and control subsystem B. Arbitrary local operations and measurements on system B and classical communication between A and B are permitted, while only incoherent operations can be applied to system A. The goal is to maximize the coherence of system A with this restricted set of operations.

**Figure 1:**
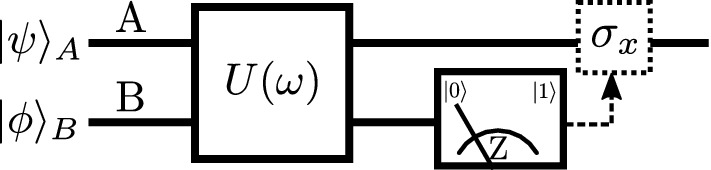
Quantum circuit of the measurement-induced quantum coherence enhancement. The target system A and control system B are coupled by a fixed Hamiltonian but the coupling strength $\omega$ can be controlled. Only incoherent operations can be applied locally to systems A and B. After the interaction with A the system B is measured in the basis of incoherent states and the measurement outcome is transmitted to A who can apply a *strictly incoherent*^1^ unitary operation $\sigma _X$ that flips the basis states $|0\rangle$ and $|1\rangle$.

In the present work we further investigate this remote control and enhancement of quantum coherence. We go beyond the paradigm of assisted coherence distillation and consider a setting depicted in Fig. 1, where we start from a product state of target system A and control system B, with limited local coherence in each subsystem. The two systems then interact via suitable coupling with a controllable coupling strength, which establishes quantum correlations between A and B. This coupling does not generate any *local coherence* from input incoherent states. Only incoherent measurements and incoherent operations are allowed locally on systems A and B. We show that for specific intersystem coupling this procedure can *deterministically* enhance the local coherence of A while fully preserving its purity, and it works for any pure control state with non-zero coherence. This measurement-based protocol can be iterated and the state of system A can be deterministically steered to a state with maximum coherence. We also show that instead of controlling the coupling strength of the interaction between the two systems, we can consider a fixed coupling strength, and impose suitable phase shift on the input control system B. We experimentally demonstrate our protocol with quantum photonic platform, where two-level quantum systems are represented by polarization states of single photons and their tunable interaction is provided by a linear optical partial SWAP gate. We develop our protocol for two-level systems and in the concluding part of the paper discuss possible extensions to higher dimensional systems.

## Results

### The protocol

We consider the quantum circuit illustrated in Fig. 1 with target qubit A and control qubit B initially prepared in pure states

1 $$\begin{aligned} |\psi \rangle _A=\cos \alpha |0\rangle +\sin \alpha |1\rangle , \quad |\phi \rangle _B=\cos \beta |0\rangle +\sin \beta |1\rangle , \end{aligned}$$

where $|0\rangle$ and $|1\rangle$ represent the basis of incoherent states of each qubit. Coherence ^1^ of pure state $|\psi \rangle$ is quantified by the entropy of probabilities of the basis states $|0\rangle$ and $|1\rangle$,

2 $$\begin{aligned} C = h(\cos ^2\alpha ), \end{aligned}$$

where $h(x)=-x\log _2(x)-(1-x)\log _2(1-x)$. The coherence is maximized for balanced superposition state $(|0\rangle +|1\rangle )/\sqrt{2}$, i.e. at $\alpha =\pi /4$. As shown in Fig. 1, the qubits A and B are coupled via a unitary operation $U=\exp (-iHt)$ induced by Hamiltonian *H*. Subsequently, the control qubit B is measured in the basis of incoherent states and a strictly incoherent operation can be applied to target qubit A depending on the result of measurement on qubit B. The goal of the protocol is to deterministically enhance the coherence of target qubit A while fully preserving its purity. In what follows, we show that this is possible for a non-trivial interaction Hamiltonian *H* that preserves the total population of levels $|1\rangle$, hence it couples only the basis states $|01\rangle$ and $|10\rangle$. Such a Hamiltonian is available for many physical systems including superconducting qubits^22,23^, trapped ions^24^ or neutral atoms^25^, and thus well motivated. More specifically, we take

3 $$\begin{aligned} H= ig(|01\rangle \langle 10|-|10\rangle \langle 01|), \end{aligned}$$

where *g* is an interaction strength. Consequently, we have

4 $$\begin{aligned} U=\left( \begin{array}{cccc} 1 &{} 0 &{} 0 &{} 0 \\ 0 &{} \cos (gt) &{} \sin (gt) &{} 0 \\ 0 &{} -\sin (gt) &{} \cos (gt) &{} 0 \\ 0 &{} 0 &{} 0 &{} 1 \end{array} \right) . \end{aligned}$$

This coupling does not generate any local coherence if the qubits A and B are initially prepared in incoherent states $\rho _A$ and $\rho _B$ (i.e., density matrices diagonal in the computational basis). By the *local coherence* of a system $\rho$ we mean a coherence *C* of its individual qubits, e.g., for an *i*-th subsystem, the local coherence is $C(\textrm{Tr}_{j\ne i}[\rho ])$. Although operation (4) generally introduces correlations between the subsystems, the subsequent partial trace prevents gaining local coherence for initially incoherent states. On the other hand, we will later show that coupling (4) increases the local coherence of initially partially coherent input qubits.

In our protocol, a nonvanishing local coherence of control qubit B represents a resource that can be used to increase the local coherence of qubit A. The protocol requires control of the effective two-qubit coupling strength $\omega =gt$. In practice, this could be achieved, e.g., by choosing the time *t* when the control system B is measured, thus controlling the effective interaction time. The optimal coupling strength $\omega$ can be determined from the requirement that the normalized output states of qubit A $|\psi _0\rangle _A$ and $|\psi _1\rangle _A$, that correspond to projection of qubit B onto $|0\rangle$ or $|1\rangle$, will possess the same coherence. This yields

5 $$\begin{aligned} \tan \omega =\frac{\tan \alpha - \cot \alpha }{\tan \beta +\cot \beta }, \end{aligned}$$

and

6 $$\begin{aligned} |\psi _0\rangle _A=\cos {\tilde{\alpha }}|0\rangle +\sin {\tilde{\alpha }}|1\rangle , \qquad |\psi _1\rangle _A=\sin {\tilde{\alpha }}|0\rangle +\cos {\tilde{\alpha }}|1\rangle , \end{aligned}$$

where

7 $$\begin{aligned} \tan {\tilde{\alpha }}=\frac{\tan \alpha \cot \beta +\cot \alpha \tan \beta }{\sqrt{\tan ^2\beta +\cot ^2\beta +\tan ^2\alpha +\cot ^2\alpha }}. \end{aligned}$$

The derivations of these equations are provided in the Supplemental Material. The state $|\psi _1\rangle _A$ can be deterministically converted to state $|\psi _0\rangle _A$ by local strictly incoherent unitary operation

8 $$\begin{aligned} \sigma _X=|0\rangle \langle 1|+|1\rangle \langle 0|. \end{aligned}$$

This operation only flips the basis states and cannot increase the coherence of the state.

**Figure 2:**
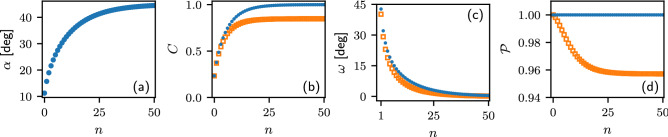
Convergence of the deterministic coherence enhancement protocol for $\alpha _0=\pi /16$ and $\beta =\pi /16$. We plot the parameter $\alpha$ specifying the state of qubit A after *n* iterations (**a**), the coherence of qubit A (**b**), the dependence of the coupling strength $\omega$ on the iteration step *n* (**c**), and the state purity $\mathscr {P}=\textrm{Tr}\left( \rho ^2\right)$ (**d**). Blue circles represent results for the protocol with measurement and feed-forward. For comparison, orange squares indicate results for a scheme without measurement.

The above protocol enhances the coherence of target qubit A for any control state $|\phi \rangle _B$ with nonvanishing coherence. Indeed, assuming $0<\beta <\pi /2$ one can prove the following strict inequalities

9 $$\begin{aligned} \min (\tan \alpha ,\cot \alpha )< \tan \tilde{\alpha } < \max (\tan \alpha ,\cot \alpha ), \end{aligned}$$

see the Supplemental Material for the proof. These inequalities imply $|\tilde{\alpha }-\pi /4|<|\alpha -\pi /4|$. This proves that the coherence of qubit A is enhanced because the angle $\alpha$ gets closer to $\pi /4$. If several copies of control state $|\phi \rangle _B$ are available, we can iterate the protocol and repeatedly apply the map $\alpha \rightarrow \tilde{\alpha }$ to asymptotically generate a state with maximal coherence in qubit A. The convergence to $\alpha =\pi /4$ is asymptotically exponentially fast. Assume $\tan \alpha =1-\epsilon$ with $\epsilon \ll 1$. Then

10 $$\begin{aligned} \tan \tilde{\alpha }\approx 1 +\frac{\cot \beta -\tan \beta }{|\tan \beta +\cot \beta |}\epsilon . \end{aligned}$$

Since

$$\begin{aligned} q=\left| \frac{\cot \beta -\tan \beta }{\tan \beta +\cot \beta }\right| <1 \end{aligned}$$

we get $\epsilon \rightarrow q\epsilon$ and an exponentially fast convergence. We can thus deterministically concentrate the coherence by the measurement and pump it to qubit A starting from several copies of control qubits $|\phi \rangle _B$ with low coherence.

**Figure 3:**
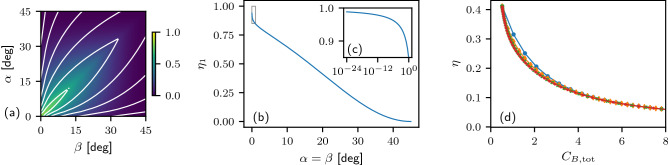
(**a**) Color-coded efficiency of a single step $\eta _1$ versus initial parameters $\alpha , \beta$, white contours mark levels 0.1–0.8 with 0.1 steps. The plot is symmetrical because we assume that $\beta \le \alpha$ and if not, we swap the qubits. (**b**,**c**) Section of (**a**) along the line $\alpha =\beta$, (**c**) is evaluated in logarithmic scale to show the convergence $\eta _1 \rightarrow 1$ in the limit of $\alpha \rightarrow 0$. (**d**) Numerically approximated overall efficiency $\eta$ ($n = 1000$, $\beta _1 = 20$ deg) is plotted versus the total coherence consumed from system B. The tested evolutions of $C_{B,(k)}$ are marked with the following colors and symbols: blue dots - $f_1$, orange diamonds - $f_2$, green ’x’ marks - $f_3$, and red crosses - $f_4$. The profile functions $f_j$ are defined in Eq. (13).

We can quantify the efficiency of coherence transfer in a single step of the protocol as a ratio of coherence gain in target qubit A to coherence consumed from control qubit B,

11 $$\begin{aligned} \eta _1 = \frac{C_{A,(1)} - C_{A,(0)}}{C_{B,(1)}}, \end{aligned}$$

where $C_{A,(0)}$ is the initial coherence of qubit A, $C_{B,(1)}$ is the coherence of qubit B consumed in the first step, and $C_{A,(1)}$ is the coherence of qubit A after the first step of the protocol. The initial coherences $C_{A,(0)}$ and $C_{B,(1)}$ are determined directly by Eq. (2) as a function of the initial parameters $\alpha$, $\beta$, and the coherence $C_{A,(1)}$ is determined with the help of the output angle $\tilde{\alpha }$ defined in Eq. (7). The color plot of the efficiency $\eta _1$ in Fig. 3a shows that a single step is maximally efficient for $\alpha = \beta$. The plots in Fig. 3b,c show that the efficiency in this regime reaches unity in the limit of low input coherence.

Let us now define the overall coherence transfer efficiency in the following way,

12 $$\begin{aligned} \eta = \lim _{n\rightarrow \infty } \frac{C_{A,(n)} - C_{A,(0)}}{C_{B,\textrm{tot}}}, \end{aligned}$$

where $C_{A,(n)}$ describes coherence of qubit A after *n* iterations of the protocol and the total coherence consumed from system B is

$$\begin{aligned} {C_{B,\textrm{tot}} = \sum _{k=1}^{n} C_{B,(k)}}, \end{aligned}$$

with $C_{B,(k)}$ being the coherence of qubit B consumed in the *k*-th iteration. In $C_{B,\textrm{tot}}$ we used additivity of coherence measure for factorized control qubits. In the case of constant $\beta$ and infinite number of iterations, $C_{B,\textrm{tot}}$ diverges, and the efficiency is zero. We have numerically investigated the case in which $\beta _k$ changes so that $C_{B,\textrm{tot}}$ converges. For simplicity, we assume that initially $\alpha = \beta$. We choose the series $\beta _k$ such that $C_{B,(k)} = C_{B,(1)}f_i(k)$, with the following choice of profile functions $f_i(k)$:

13 $$\begin{aligned} f_1(k) = \Theta (\kappa -(k-1)), \qquad f_2(k) =\max (1-\kappa (k-1), 0), \qquad f_3(k) = e^{-\kappa (k-1)}, \qquad f_4(k) =\frac{1}{1+(\kappa (k-1))^2}, \end{aligned}$$

where $\Theta$ is the Heaviside step function. We then sweep the parameter $\kappa$ which controls the total coherence consumption $C_{B,\textrm{tot}}$ and evaluate the overall efficiency (12) as a function of the consumed coherence. The results in Fig. 3d suggest that the efficiency mainly depends on the total spent coherence rather than on the exact evolution of $C_{B,(k)}$, although is seems that the step-function evolution of $C_{B,(k)}$ provides a slightly better efficiency for low $C_{B,\textrm{tot}}$. The maximum of $\eta$ is determined by the efficiency of the first step, which reaches unity in the limit of low input coherence. The efficiency will asymptotically decrease to zero for large amounts of consumed coherence $C_{B,\textrm{tot}}$.

Note that the conditional application of the operation $\sigma _X$ to qubit A is not really necessary for deterministic coherence concentration. One can instead keep track of the measurement outcomes on qubits B and adapt the coupling strength $\omega$ at each step accordingly. If the measurement outcome on qubit B is ’1’, then we instead of $\sigma _x$ application select the next $\omega ' = -\omega$ where $\omega$ is selected using Eq. (5). This choice satisfies the condition on equal coherence of output conditional states when the input qubit A has been flipped. The detailed derivation is provided in Supplemental Material. The protocol will then deterministically converge to $\alpha =\pi /4$. Typical behavior of the protocol is illustrated in Fig. 2. Note that the coupling strength $\omega$ decreases at each iteration of the protocol and asymptotically vanishes.

For comparison, we provide in Fig. 2 also results of a simpler protocol that does not involve any measurement and feed-forward. In this latter scheme, we throw away the qubit B after the interaction and we numerically optimize the coupling strength $\omega$ at each iteration to maximize the coherence of the output state of qubit A. Note that the state of qubit A becomes mixed in this process as illustrated in Fig. 2d. Therefore *C* is evaluated using the general formula for coherence of a mixed state^1^

14 $$\begin{aligned} C(\rho )=S(\Delta (\rho )) -S(\rho ), \end{aligned}$$

where $S(\rho )=-\textrm{Tr}(\rho \log _2\rho )$, and $\Delta (\rho )=\rho _{00}|0\rangle \langle 0|+\rho _{11}|1\rangle \langle 1|$ is the density matrix of the completely dephased state. The protocol without measurement does not converge to a maximally coherent state and the coherence saturates at an asymptotic value that is strictly smaller than 1, see Fig. 2b. This illustrates the importance and usefulness of the measurement and feed-forward that enable us to control and enhance the coherence while fully preserving the purity of the target qubit.

Instead of controlling the coupling strength $\omega$, we can also control the concentration of quantum coherence by applying a phase shift $\varphi$ to input qubit B, which yields the input control state $\cos \beta |0\rangle +e^{i\varphi }\sin \beta |1\rangle$. This latter approach is less universal, because it works only for a restricted range of input states and coupling strengths, but is appealing because the control of two-qubit interaction is replaced with local control of qubit B. We illustrate this protocol for a maximally entangling two-qubit gate *U* obtained at $\omega =\pi /4$. We again require that the two conditional output states of qubit A $|\psi _{0,1}\rangle$ possess the same coherence. This yields an expression for the phase shift $\varphi$,

15 $$\begin{aligned} \cos \varphi =\frac{1}{2}\frac{\tan ^2\alpha +\tan ^2\beta -\cot ^2\alpha -\cot ^2\beta }{\tan \alpha \tan \beta +\cot \alpha \cot \beta }, \end{aligned}$$

the derivation is provided in the Supplemental Material. The condition $|\cos \varphi |\le 1$ defines the range of $\alpha$ and $\beta$ for which the protocol works. In particular, this condition is always satisfied if $\alpha =\beta$. Numerical calculations confirm that if the phase shift (15) exists, then the protocol enhances the coherence of qubit A. Besides the bit flip $\sigma _X$ the two conditional output states of qubit A will differ also by some phase shift $\delta$ of the amplitude of state $|1\rangle$ that should be compensated by a local strictly incoherent operation $\exp (i\delta \sigma _Z)$, where $\sigma _Z=|0\rangle \langle 0|-|1\rangle \langle 1|$, or tracked and taken into account in the iterative version of the protocol. For $\omega =\pi /4$ we find that the iterative protocol with fixed $\beta$ and initial point $\alpha _0=\beta$ will converge to state with maximum coherence provided that $\pi /8<\beta <3\pi /8$.

### Experimental setup

**Figure 4:**
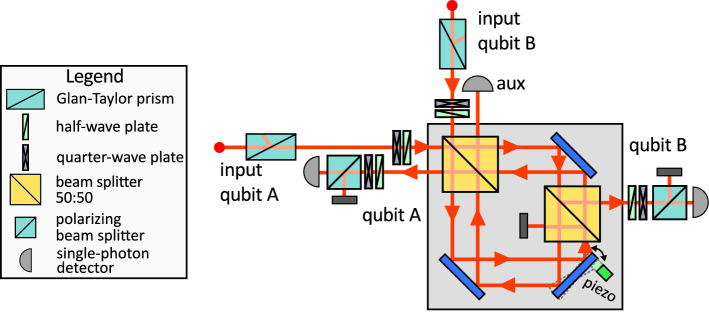
Experimental setup. The Mach–Zehnder interferometer is folded into displaced Sagnac interferometer. Polarization states of single photons are prepared and analyzed with the use of waveplates, polarizing beam splitters and Glan-Taylor prisms. Photons are detected by silicon avalanche photodiodes operating in the Geiger mode. The auxiliary detector is used only for the tuning of the interferometric phase.

We have experimentally tested the proposed protocols with a quantum photonic setup^26,27^, where qubits are encoded into polarization states of single photons. The interaction between the two qubits is provided by a unitary partial-SWAP gate

16 $$\begin{aligned} U_{\textrm{PSWAP}}=\Pi _{+}+e^{i2\omega }\Pi _{-}, \end{aligned}$$

where $\Pi _{-}=|\Psi _{-}\rangle \langle \Psi _{-}|$ is the projector onto the anti-symmetric singlet Bell state $|\Psi _{-}\rangle =\frac{1}{\sqrt{2}}(|01\rangle -|10\rangle )$, $\Pi _{+}=I-\Pi _{-}$ is the projector onto the three-dimensional symmetric subspace of two-qubits, and *I* denotes the identity operator. The partial SWAP gate induces the same coupling of states $|01\rangle$ and $|10\rangle$ as the Hamiltonian (3) up to local phase shifts that can be taken into account and do not affect the performance of the presented protocol. See Supplemental Material for the detailed analysis. Design of the linear optical partial SWAP gate^28^ is described in Methods section.

Detailed experimental setup is depicted in Fig. 4. Its core that implements the partial SWAP gate is formed by a displaced Sagnac interferometer, which ensures the inherent passive interferometric stability of the setup^29^. Correlated photon pairs are generated in the process of spontaneous parametric down-conversion in a nonlinear crystal pumped by a laser diode (not shown in the figure). The two photons are spatially separated at a polarizing beam splitter and guided to the depicted experimental setup. Polarization states of photons are prepared and controlled with half- and quarter-wave plates and Glan-Taylor prisms. Then the photons enter the central interferometer which implements the partial SWAP gate. At the output, the photons are detected by single-photon avalanche photodiodes. With this compact and inherently stable setup we have implemented the partial-SWAP gate with the unprecedently high gate fidelity that exceeded 0.97 for all tested coupling strengths $\omega$ in the interval $[0,\pi /2]$.

The photonic platform employed in our experiment provides a convenient testbed for proof-of-principle demonstration and verification of the proposed protocol for controlled enhancement of quantum coherence. Although the coherence of polarization states of single photons could be easily manipulated with the waveplates, we do not use the waveplates for such purpose in the main part of our experiment. We emphasize that we utilize the waveplates solely to prepare the input states and to set the measurement basis for the characterization of the output states. The partial SWAP operation that forms the core of the demonstrated protocol is implemented with a Mach–Zehnder interferometer that does not contain any waveplates. Our results reported below thus confirm the functioning of the protocol, which is applicable to any physical system, including those where the coherence changing-operations can be more experimentally demanding and costly than incoherent operations.

### Experimental results

We have first experimentally probed a single step of the coherence enhancement procedure. In order to compensate for the additional phase shifts induced by the partial SWAP gate, the qubit A is prepared in state $\cos \alpha |0\rangle +i\sin \alpha |1\rangle$ while the control qubit B was prepared in state $\cos \beta |0\rangle +\sin \beta |1\rangle$.

In this measurement we have probed the symmetric scenario where both qubits A and B initially have the same coherence, $\alpha =\beta$. The two-qubit coupling strength $\omega$ is set according to Eq. (5). We perform full quantum tomography of the output two-qubit state, reconstruct the density matrix by likelihood maximization^30^, and extract from it (non-normalized) density matrices $\rho _{A0}$ and $\rho _{A1}$ corresponding to projection of qubit B onto the basis states $|0\rangle$ and $|1\rangle$, respectively. We then apply a correcting phase shift $2\omega$ together with the conditional bit flip $\sigma _X$ to the reconstructed density matrix $\rho _{A1}$ and obtain the overall output state of qubit A, $\rho _A=\rho _{A0}+\sigma _X e^{-i\omega \sigma _Z}\rho _{A1}e^{i\omega \sigma _Z}\sigma _{X}$. Alternatively, we can choose only the subset of the two-qubit coincidences that correspond to projections of qubit B onto the computational basis states, and from this restricted data set we directly reconstruct the single-qubit density matrices $\rho _{A0}$ and $\rho _{A1}$. These two procedures yield very similar results and in what follows we report data obtained with the former procedure.

**Figure 5:**
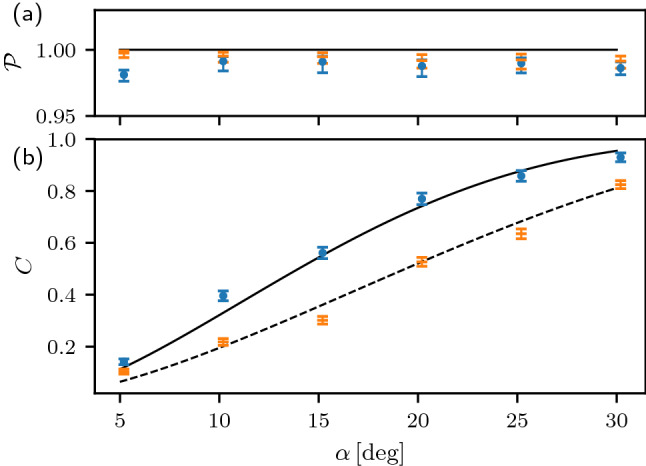
Experimental results for a single step of the coherence enhancement protocol with identical input states of qubits A and B, $\alpha =\beta$. The experimentally determined purity $\mathscr {P}$ (**a**) and coherence *C* (**b**) of input (orange horizontal dashes) and output (blue circles) state of qubit A are plotted for 6 different input states. The solid and dashed lines indicate theoretical predictions (they coincide for the purity $\mathscr {P}$).

The experimental results are displayed in Fig. 5 for six different values of $\alpha$. We plot in the figure the coherence of the state *C* as well as the state purity $\mathscr {P}=\textrm{Tr}(\rho ^2)$. Since the experimentally determined states are not exactly pure, we use the general expression for coherence of a mixed state (14). For reference, the curves in Fig. 5 specify the theoretical prediction for an ideal pure-state protocol. We can see that the experimental data closely follow the theoretical expectation. The protocol enhances the local coherence of qubit A while maintaining its very high purity. The input state is practically perfectly pure, with $\mathscr {P}>0.992$ for all $\alpha$ considered, while the output state becomes slightly mixed. This can be attributed mainly to the imperfections of the two-qubit partial SWAP gate, such as residual phase fluctuations in the interferometer and an imperfect visibility of two-photon interference.

Here and in the rest of our paper, the error-bars represent one standard deviation and were obtained using parametric bootstrapping. With the knowledge of reconstructed states, measurement operators, mean count-rate in the tomogram, and under the assumption of the Poissonian distribution of measured coincidence number, we generated 1000 tomograms, processed them the same way as the original tomograms, obtaining a set for each quantity of interest (e.g. coherence, purity). We evaluated the standard deviation of this set. For quantities of interest near its theoretical boundary, purity in this case, we instead found 0.158 and 0.84 quantiles and used them to plot asymmetrical error-bars, in which lies 68.2% of all samples, equivalently to one standard deviation.

**Figure 6:**
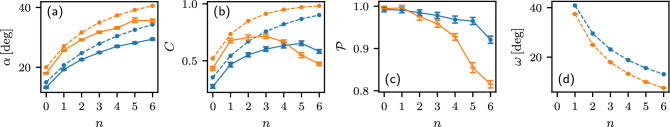
Experimental test of iterative coherence enhancement. The effective angle $\alpha$ (**a**), coherence (**b**) and purity (**c**) of qubit A and the coupling strength $\omega$ (**d**) are plotted in dependence on the number *n* of steps of the protocol. The results are presented for two different inputs $\alpha _0=\beta =15^\circ$ (blue) and $\alpha _0=\beta =20^\circ$ (orange). Symbols represent experimental data, solid lines guide the eye, and dashed lines indicate theoretical predictions. Data at $n = 0$ represent the reference input state.

Having verified the functioning of a single step of the protocol, we now proceed to experimental test of the iterative coherence enhancement scheme. At each step, we determine the output density matrix $\rho _A$ of qubit A and use it as an input state of the next step of the protocol, while keeping the state of qubit B (i.e. the angle $\beta$) fixed at each step. The suitable coupling strength $\omega$ is at each step again determined from Eq. (5), where the angle $\alpha$ is chosen according to the theoretical prediction for ideal pure state protocol, depicted in Fig. 2. We prepare a mixed polarization state $\rho _A$ of a single photon by preparing a statistical mixture of the two eigenstates of $\rho _A$ with weights equal to the corresponding eigenvalues. Such preparation allows us to study the noise accumulation effect in the protocol, in contrast to the case of pure state preparation only. We start the iterative protocol from a symmetric input, $\alpha _0=\beta$. In Fig. 6 we plot the experimental results for two different initial coherences, $\alpha _0=15^{\circ }$ and $\alpha _0=20^{\circ }$.

The figure displays the coherence and purity of the state after each step of the protocol, together with the effective angle $\alpha$, and the utilized two-qubit coupling strength $\omega$. The angle $\alpha$ was determined from the dominant eigenstate $|a_1\rangle$ of $\rho _A$ and characterizes the coherence of $|a_1\rangle$. Therefore, the angle $\alpha$ does not contain any information about the purity of the state, it rather describes the eigenbasis of the output states. We can write $\rho _A=(2a_1-1)|a_1\rangle \langle a_1|+(1-a_1)I$, where $a_1$ is the maximum eigenvalue of $\rho _A$, *I* denotes the identity operator, and *I*/2 represents a maximally mixed state. The effective angle $\alpha$ increases at each step and closely follows the theoretical prediction, i.e. the dominant eigenstate $|a_1\rangle$ of the output state $\rho _A$ evolves according to the protocol. However, simultaneously the noise and imperfections accumulate and the state purity $\mathscr {P}=\textrm{Tr}[\rho _A^2]$ is reduced after each step of the protocol, as shown in Fig. 6c. The loss of purity reduces the coherence of the output state $\rho _A$ and at a certain point, this effect outweighs the gain of coherence induced by the evolution of the dominant eigenstate $|a_1\rangle$. Therefore, the coherence of qubit A starts to decrease after some number of iterations of the protocol, see Fig. 6b. Specifically, for $\alpha _0=15^\circ$ we observe that the coherence increases up to the 5th step of the protocol while for $\alpha _0=20^\circ$ the coherence of qubit A reaches its maximum already at the 3rd step and then drops down. The data in Fig. 6 thus illustrate the sensitivity of the quantum coherence manipulation protocol to noise and imperfections. Thanks to the very high fidelity of our linear optical partial SWAP gate, we were able to observe the improvement of coherence up to 5 iterations of the protocol.

The difference in coherence maxima positions in Fig. 6b is related mainly to the partial SWAP gate implementation. Computational basis states $|01\rangle$ and $|10\rangle$ are coupled by the partial SWAP gate, and the coupling strength $\omega$ is determined by the interferometric phase $\varphi = 2\omega$. Therefore, the output populations of $|01\rangle$ and $|10\rangle$ are sensitive to $\varphi$ and vulnerable to phase noise. These populations increase as the parameters $\alpha$ and $\beta$ get closer to $\pi /4$, and therefore the protocol becomes more vulnerable to dephasing in this limit. Moreover, the phase misalignment breaks the condition $|\psi _0\rangle _A = \sigma _x |\psi _1\rangle _A$, which increases the mixedness of the output state. Also this effect becomes more pronounced when $\alpha$ and $\beta$ get closer to $\pi /4$, because then the probabilities of the two measurement outcomes on qubit B become more balanced.

**Figure 7:**
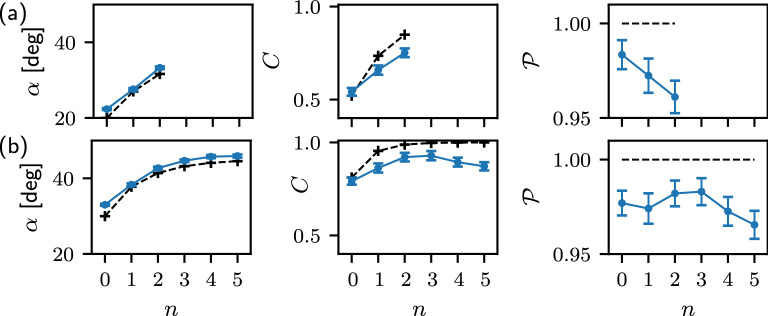
Experimental results for iterative protocol with a fixed coupling strength $\omega =\pi /4$ and control exercised by phase shifts applied to input qubit B. The coherence, purity and effective angle $\alpha$ of qubit A are plotted for two different inputs $\alpha _0=\beta =20^\circ$ (**a**) and $\alpha _0=\beta =30^\circ$ (**b**). Data at $n = 0$ represent the reference input state. Blue dots are experimental data, black ’+’ marks show the theoretical prediction for comparison. The lines are to guide the eye.

Finally, we have experimentally tested the alternative protocol, where the coupling strength $\omega$ is fixed and at each step of the protocol we adjust the phase of qubit B to enhance the coherence of qubit A. Experimental results for this protocol are displayed in Fig. 7. We set $\omega =\pi /4$ hence we employ the maximally entangling $\sqrt{\textrm{SWAP}}$ gate as considered in the preceding theoretical analysis. The left panels show results for $\alpha _0=20^\circ$, when this protocol cannot be iterated to infinity and terminates after second step, because the phase shift (15) that should be applied to the control qubit B does not exist anymore. In Fig. 7b we present results for $\alpha _0=30^\circ$. In this case the protocol can be arbitrarily iterated and in theory should converge to a maximally coherent state. In practice, we observe that the coherence grows up to the third iteration and then it begins to moderately decrease again as the noise accumulates.

## Discussion

We have presented and experimentally tested a novel protocol for control and enhancement of quantum coherence under a restricted set of operations that include a local strictly incoherent operations and measurements, feed-forward, and a fixed interaction Hamiltonian with a tunable coupling strength. We have observed that the quantum coherence of the target system can be remotely deterministically controlled and steered to a maximally coherent state within this setting. The considered set of operations is practically motivated, because the strictly incoherent operations and measurements are usually easy to implement and also the considered interaction Hamiltonian (3) is physically well motivated and available for many experimental systems and platforms such as superconducting qubits, trapped ions and neutral atoms^22–25^.

While we have presented the protocol for two-dimensional systems (qubits), extension to higher-dimensional systems is possible. Consider interaction Hamiltonian $H_{jk}=ig(|jk\rangle \langle kj|-|kj\rangle \langle jk|)$. Following the above protocol and utilizing a control system B prepared in superposition of states $|j\rangle$ and $|k\rangle$ one can enhance the quantum coherence of target system in a two-dimensional subspace spanned by $|j\rangle$ and $|k\rangle$. One can then apply a unitary permutation operation $U_\pi =\sum _j|\pi (j)\rangle \langle j|$ to the target system to address a different subspace and repeat the whole procedure to drive the state of the target system A towards the maximally coherent state. One can also consider variants of this protocol, where one can switch on and off couplings of different pairs of quantum levels $|j\rangle$ and $|k\rangle$ or even simultaneously switch on several such elementary couplings. A detailed study of these scenarios will be the subject of future work.

## Methods

**Figure 8:**
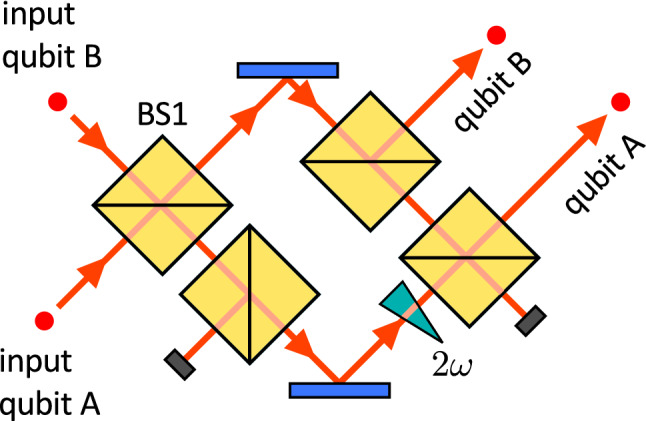
Linear optical partial SWAP gate^28^. A Mach–Zehnder interferometer is formed by two balanced beam splitters. Two additional balanced beam splitters are inserted inside the interferometer. The interaction strength $\omega$ is determined by the relative phase shift between the interferometer arms. Successful gate operation is indicated by coincidence detection of a single photon in each of the two output gate ports indicated in the figure.

### Linear optical partial SWAP gate

The linear optical partial-SWAP gate is schematically illustrated in Fig. 8. The gate is formed by a balanced interferometer with an additional balanced beam splitter inserted into each of its arms^28^. The coupling strength $\omega$ is controlled by the phase shift between the two interferometer arms and is fully tunable. The gate operation is based on two-photon interference at a balanced beam splitter. If the input photons are in a symmetric state, they bunch at the first balanced beam splitter and must propagate through the upper interferometer arm to reach the designated gate outputs. On the other hand, if the photons are initially at the anti-symmetric singlet state, they remain antibunched after interference at BS1 and each photon propagates in one arm of the interferometer, which imposes the phase shift $2\omega$ between the symmetric and antisymmetric states of the two qubits. The gate operates in the coincidence basis and its successful application is heralded by coincidence detection of a single photon in each of the two gate output ports indicated in Fig. 8. Similarly to other linear optical quantum gates^26^, the gate is probabilistic and its theoretical success probability is $\frac{1}{8}$ irrespective of the coupling strength $\omega$. In the experiment, we automatically post-select the successful events by measuring two-photon coincidences between the two output ports of the gate. Note that this probabilistic nature of the linear optical partial SWAP gate does not preclude testing of our deterministic protocol, because it only reduces the data acquisition rate, but upon success it realizes the required quantum circuit that could in principle be implemented deterministically on other platforms.

The interferometric phase is controlled by a piezo element coupled to a mirror in the interferometer. To set the phase, we first block the input beam B and measure the single-photon counts at the outputs of the interferometer. A computer program then controls the piezo voltage to reach the desired set point. We have separately tested the program and observed the deviation of phase setting lesser than 1.1 degrees RMS. This automatic method sometimes fails due to noise and hysteresis. In the experiment, we have always checked whether the adjustment procedure succeeded, and in the case of failure, we have repeated the adjustment. Detailed test results are provided in the Supplemental Material.

## Supplementary Information

Supplementary Information.

## Data Availability

The data that support the findings of this study are available from the corresponding author upon reasonable request. The code used for generation of the presented numerical results and the code used to process the experimental data are available from the corresponding author upon reasonable request.
