# Supplementary material for: Deterministic controlled enhancement of local quantum coherence
Source: Sci Rep. 2022 Dec 27;12:22455. doi: 10.1038/s41598-022-26450-1 (PMC9794828; doi:10.1038/s41598-022-26450-1)
Supplement: Supplementary file 1 — Supplementary Information. [file 41598_2022_26450_MOESM1_ESM.pdf]

Supplemental material:
Deterministic controlled enhancement of local quantum coherence

Nikola Horová[1], Robert Stárek[1,*], Michal Mičuda[1], Michal Kolář[1], Jaromír Fiurášek[1], and Radim Filip[1]

[1]Department of Optics, Palacký University, Faculty of Science, 17. listopadu 1192/12, 779 00 Olomouc, Czech Republic
[*]starek@optics.upol.cz

1 Derivation of Equations (5) and (7)

Input state $|\psi\rangle_A \otimes |\phi\rangle_B$ defined in the main text in Eq. (1) as $|\psi\rangle_A = \cos\alpha|0\rangle + \sin\alpha|1\rangle$, $|\phi\rangle_B = \cos\beta|0\rangle + \sin\beta|1\rangle$, is transformed by partial swap operator (4)

$$U = \begin{pmatrix} 1 & 0 & 0 & 0 \\ 0 & \cos(gt) & \sin(gt) & 0 \\ 0 & -\sin(gt) & \cos(gt) & 0 \\ 0 & 0 & 0 & 1 \end{pmatrix}$$

into state

$$|\psi'\rangle_{AB} = \begin{pmatrix} \cos(\alpha)\cos(\beta) \\ \cos(\alpha)\cos(\omega)\sin(\beta) + \sin(\alpha)\cos(\beta)\sin(\omega) \\ \sin(\alpha)\cos(\beta)\cos(\omega) - \cos(\alpha)\sin(\beta)\sin(\omega) \\ \sin(\alpha)\sin(\beta) \end{pmatrix}, \tag{S1}$$

which is then transformed by computational basis projections on part B into two conditional states

$$|\psi_0\rangle_A = (\mathbb{I}_A \otimes \langle 0|_B)\,|\psi'\rangle_{AB} = \begin{pmatrix} \cos(\alpha)\cos(\beta) \\ \sin(\alpha)\cos(\beta)\cos(\omega) - \cos(\alpha)\sin(\beta)\sin(\omega) \end{pmatrix}, \tag{S2}$$

$$|\psi_1\rangle_A = (\mathbb{I}_A \otimes \langle 1|_B)\,|\psi'\rangle_{AB} = \begin{pmatrix} \cos(\alpha)\sin(\beta)\cos(\omega) + \sin(\alpha)\cos(\beta)\sin(\omega) \\ \sin(\alpha)\sin(\beta) \end{pmatrix}, \tag{S3}$$

where $\mathbb{I}$ is the identity operator. The requirement for equal coherence of states $|\psi_0\rangle_A$ and $|\psi_1\rangle_A$ is equivalent to the requirement for equal population imbalance, i.e., either $\frac{|\langle 1|\psi_0\rangle_A|}{|\langle 0|\psi_0\rangle_A|} = \frac{|\langle 1|\psi_1\rangle_A|}{|\langle 0|\psi_1\rangle_A|}$ or $\frac{|\langle 1|\psi_0\rangle_A|}{|\langle 0|\psi_0\rangle_A|} = \frac{|\langle 0|\psi_1\rangle_A|}{|\langle 1|\psi_1\rangle_A|}$. In our case, we satisfy the latter equality condition. If the condition is met, $|\psi_1\rangle_A$ can be transformed into $|\psi_0\rangle_A$ using strictly incoherent σ_X operation. This equality condition yields equation

$$\cos\omega\tan\alpha - \sin\omega\tan\beta = \pm(\cos\omega\cot\alpha + \sin\omega\cot\beta) \tag{S4}$$

which can be solved for $\tan\omega$. The solution for the positive branch is directly Equation (5)

$$\tan\omega = \frac{\tan\alpha - \cot\alpha}{\tan\beta + \cot\beta}$$

and the other solution is

$$\tan\omega_- = \frac{1}{(\tan\beta + \cot\beta)\sin\alpha\cos\alpha}. \tag{S5}$$

Solution (5) leads to the conditional states in the form $|\psi_{0+}\rangle_A = \cos\tilde{\alpha}|0\rangle + \sin\tilde{\alpha}|1\rangle$ and $|\psi_{1+}\rangle_A = \sin\tilde{\alpha}|0\rangle + \cos\tilde{\alpha}|1\rangle$, while solution (S5) leads to conditional states $|\psi_{0-}\rangle_A = |\psi_{0+}\rangle_A$ and $|\psi_{1-}\rangle_A = \sin\tilde{\alpha}|0\rangle - \cos\tilde{\alpha}|1\rangle$. The additional π phase shift could be compensated for using conditional σ_Z operation. Both solutions for ω are valid, but we use only solution (5) for simplicity.

We calculate the ratio $\frac{|\langle 1|\psi_0\rangle_A|}{|\langle 0|\psi_0\rangle_A|} = \tan\tilde{\alpha}$ with the knowledge of the conditional states (S2) and (S3) and replace the arising $\tan\omega$ terms with the expression for the optimal coupling strength (5) to obtain relation (7),

$$\tan\tilde{\alpha} = \frac{\tan\alpha\cot\beta + \cot\alpha\tan\beta}{\sqrt{\tan^2\beta + \cot^2\beta + \tan^2\alpha + \cot^2\alpha}}.$$

2 Proof of inequalities leading to convergence

Let us now prove the inequalities (9)

$$\min(\tan\alpha, \cot\alpha) < \tan\tilde{\alpha} < \max(\tan\alpha, \cot\alpha).$$

We assume $0 < \beta < \pi/2$. We begin with the case $0 < \alpha < \pi/4$, then inequalities (8) take form $\tan\alpha < \tan\tilde{\alpha}$ and $\tan\tilde{\alpha} < \cot\alpha$. Let us remind the expression (7) which describes the population balance of qubit A after a step of the protocol,

$$\tan\tilde{\alpha} = \frac{\tan\alpha\cot\beta + \cot\alpha\tan\beta}{\sqrt{\tan^2\beta + \cot^2\beta + \tan^2\alpha + \cot^2\alpha}}.$$

In the first inequality, we substitute $\tan\tilde{\alpha}$ with the expression (7) and after some algebra we obtain

$$\tan^4\alpha + \tan^2\beta\left(\tan^2\alpha - \cot^2\alpha\right) < 1. \tag{S6}$$

The term $\left(\tan^2\alpha - \cot^2\alpha\right)$ is negative for $0 < \alpha < \pi/4$, and also $\tan^2\beta > 0$ and $0 < \tan^4\alpha < 1$. Therefore the inequality is valid. We proceed with the inequality $\tan\tilde{\alpha} < \cot\alpha$ which transforms into

$$1 < \cot^4\alpha + \cot^2\beta(\cot^2\alpha - \tan^2\alpha).$$

The term $(\cot^2\alpha - \tan^2\alpha)$ is always positive for $0 < \alpha < \pi/4$, $\cot^4\alpha > 1$, $\cot^2\beta > 0$ holds, and therefore also the second inequality is valid. For $\pi/2 > \alpha > \pi/4$ the inequalities (8) read $\cot\alpha < \tan\tilde{\alpha}$ and $\tan\tilde{\alpha} < \tan\alpha$. We again use relation (6) to transform the first inequality into

$$\cot^4\alpha + \cot^2\beta\left(\cot^2\alpha - \tan^2\alpha\right) < 1 \tag{S7}$$

and the second one into

$$1 < \tan^4\alpha - \tan^2\beta\left(\cot^2\alpha - \tan^2\alpha\right). \tag{S8}$$

For $\pi/4 < \alpha < \pi/2$, $\cot^4\alpha < 1$, $\tan^4\alpha > 1$ and the term $\left(\cot^2\alpha - \tan^2\alpha\right)$ is negative, therefore we see that both inequalities are valid. In the remaining case of $\alpha = \pi/4$, where $\tan\alpha = \cot\alpha = 1$ we see that $\tan\tilde{\alpha} = 1$ and identify $\alpha = \pi/4$ as a fixed point of the contraction mapping performed by the protocol, concluding the proof. We did not discuss the case of negative α but the proof is very similar and straightforward. The case of negative α can be converted to the case of positive α just by applying π phase shift on the first input qubit.

3 Replacement of conditional σ_X operation with conditional choice of coupling strength

In a single step of the protocol, input state $|\psi\rangle_A \otimes |\phi\rangle_B$ defined in Eq. (1) transforms into a conditional state $|\psi_j\rangle_A = \cos(\tilde{\alpha}_j)|0\rangle + \sin(\tilde{\alpha}_j)|1\rangle$ where $j \in \{0; 1\}$ is the outcome of computational basis measurement on qubit B, and

$$\tan\tilde{\alpha}_0 = \frac{\tan\alpha\cot\beta + \cot\alpha\tan\beta}{\sqrt{\tan^2\beta + \cot^2\beta + \tan^2\alpha + \cot^2\alpha}}, \tag{S9}$$

$$\tilde{\alpha}_1 = \pi/2 - \tilde{\alpha}_0, \tag{S10}$$

and α, β are input state parameters. Clearly, the coherence of $|\psi_0\rangle_A$ and $|\psi_1\rangle_A$ are equal.

Instead of using conditional σ_X operation, we can keep track of α_j and use Eq. (5) to determine the optimal coupling strength for the next iteration, i.e.,

$$\tan\omega_j = \frac{\tan\tilde{\alpha}_j - \cot\tilde{\alpha}_j}{\tan\beta + \cot\beta}. \tag{S11}$$

Here we introduced index j to emphasize the dependence of the next coupling strength on the measurement outcome j and consequently on parameter $\tilde{\alpha}_j$. This choice guarantees the equal coherence of conditional states in the next iteration because we used Eq. (5) that fulfills this condition of equal coherence.

The relation between ω_0 and ω_1 reads

$$\tan\omega_1 = \frac{\tan\tilde{\alpha}_1 - \cot\tilde{\alpha}_1}{\tan\beta + \cot\beta} = \frac{\cot\tilde{\alpha}_0 - \tan\tilde{\alpha}_0}{\tan\beta + \cot\beta} = -\tan\omega_0 \tag{S12}$$

and is the consequence the relation between α_0 and α_1 described by Eq. (S10).

4 Derivation of Equation (15)

We consider fixed coupling $\omega = \pi/4$. Here, we show how to achieve equal coherence of the two conditional output states by tuning the phase of input qubit B. Such tuning allows using the protocol with fixed coupling.

Input state

$$|\psi_A\rangle \otimes |\phi_B\rangle = \begin{pmatrix} \cos(\alpha)\cos(\beta) \\ \cos(\alpha)\sin(\beta)e^{i\varphi} \\ \sin(\alpha)\cos(\beta) \\ \sin(\alpha)\sin(\beta)e^{i\varphi} \end{pmatrix} \tag{S13}$$

is transformed by operation (4) with fixed $\omega = \pi/4$ into

$$|\psi'\rangle_{AB} = \begin{pmatrix} \cos(\alpha)\cos(\beta) \\ \frac{1}{\sqrt{2}}\left(\sin(\alpha)\cos(\beta) + \cos(\alpha)\sin(\beta)\right)e^{i\varphi} \\ \frac{1}{\sqrt{2}}\left(\sin(\alpha)\cos(\beta) - \cos(\alpha)\sin(\beta)\right)e^{i\varphi} \\ e^{i\varphi}\sin(\alpha)\sin(\beta) \end{pmatrix}. \tag{S14}$$

The corresponding non-normalized conditional states are

$$|\psi_0\rangle_A = \begin{pmatrix} \cos(\alpha)\cos(\beta) \\ \frac{1}{\sqrt{2}}\left(\sin(\alpha)\cos(\beta) - \cos(\alpha)\sin(\beta)e^{i\varphi}\right) \end{pmatrix}, \tag{S15}$$

$$|\psi_1\rangle_A = \begin{pmatrix} \frac{1}{\sqrt{2}}\left(\cos(\alpha)\sin(\beta)e^{i\varphi} + \sin(\alpha)\cos(\beta)\right) \\ \sin(\alpha)\sin(\beta)e^{i\varphi} \end{pmatrix}. \tag{S16}$$

The requirement for equal coherence of the conditional states is equivalent to equal population imbalance, as we have discussed in Sec. 1 of this supplement. Condition

$$\frac{|\langle 1|\psi_0\rangle_A|^2}{|\langle 0|\psi_0\rangle_A|^2} = \frac{|\langle 0|\psi_1\rangle_A|^2}{|\langle 1|\psi_1\rangle_A|^2}, \tag{S17}$$

transforms into

$$\tan^2\alpha + \tan^2\beta - 2\tan\alpha\tan\beta\cos\varphi = \cot^2\alpha + \cot^2\beta + 2\cot\alpha\cot\beta\cos\varphi. \tag{S18}$$

We solve this condition for $\cos(\varphi)$ to obtain (15),

$$\cos\varphi = \frac{1}{2}\frac{\tan^2\alpha + \tan^2\beta - \cot^2\alpha - \cot^2\beta}{\tan\alpha\tan\beta + \cot\alpha\cot\beta}.$$

When the condition is met, $\sigma_X|\psi_1\rangle_A$ is equivalent to $|\psi_0\rangle_A$ in terms of population. The conditional output states $|\psi_0\rangle_A$ and $|\psi_1\rangle_A$ are locally phase-shifted by δ_0 and δ_1, respectively. The value of these phase shifts is a non-trivial function of α and β but we can compensate for them by conditional application of phase gates. Because $\cos\varphi$ is an even function, both $\pm|\varphi|$ satisfies the condition (S17). In the case of negative φ, the population ratio remains the same as in the previous solution, but the additional phases have the opposite sign, $\delta_{0-} = -\delta_0$ and $\delta_{1-} = -\delta_1$.

5 Equivalence of partial swap gate with operation (4)

Partial swap operator is defined as $U_{\mathrm{PSWAP}} = \Pi_+ + \exp(i2\omega)\Pi_-$, where $\Pi_- = |\Psi_-\rangle\langle\Psi_-|$ is the projector onto the anti-symmetric singlet Bell state $|\Psi_-\rangle = \frac{1}{\sqrt{2}}(|01\rangle - |10\rangle)$, $\Pi_+ = \mathbb{I} - \Pi_-$ is the projector onto the three-dimensional symmetric subspace of two qubits, and $\mathbb{I}$ denotes the identity operator. In the matrix form, the operator reads

$$U_{\mathrm{PSWAP}} = \begin{pmatrix} 1 & 0 & 0 & 0 \\ 0 & e^{i\omega}\cos\omega & -ie^{i\omega}\sin\omega & 0 \\ 0 & -ie^{i\omega}\sin\omega & e^{i\omega}\cos\omega & 0 \\ 0 & 0 & 0 & 1 \end{pmatrix} \tag{S19}$$

One can easily check the unitarity of the operator by evaluating $U^\dagger_{\mathrm{PSWAP}} U_{\mathrm{PSWAP}}$ and seeing that this product is equal to the identity matrix. Operator U_{PSWAP} clearly differs from operator (3). Albeit this difference, we will now show that U_{PSWAP} is equivalent to (3) in the context of the presented protocol.

We assume additional fixed local $\pi/2$ phase shift on input qubit A, then the input qubit A reads $|\psi\rangle_A = \cos(\alpha)|0\rangle + i\sin(\alpha)|1\rangle$. After application of U_{PSWAP} and measurement of qubit B in computational basis we obtain the conditional states

$$|\psi_0\rangle_A = \begin{pmatrix} \cos\alpha\cos\beta \\ ie^{i\omega}(\sin\alpha\cos\beta\cos\omega - \cos\alpha\sin\beta\sin\omega) \end{pmatrix} \tag{S20}$$

and

$$|\psi_1\rangle_A = \begin{pmatrix} e^{i\omega}(\cos\alpha\sin\beta\cos\omega + \sin\alpha\cos\beta\sin\omega) \\ i\sin\alpha\sin\beta \end{pmatrix}. \tag{S21}$$

By comparing these states to the original conditional states (S2) and (S3) we see that they differ by phase factors only. We can compensate the phase factor $i\exp(i\omega)$ in conditional state $|\psi_0\rangle_A$ by applying a fixed local phase gate $V = \exp(i\sigma_Z(\omega/2 + \pi/4))$. The same gate V also transforms conditional state $|\psi_1\rangle_A$ into

$$|\psi_1\rangle_A = \begin{pmatrix} \cos\alpha\cos\omega\sin\beta + \cos\beta\sin\alpha\sin\omega \\ \sin\alpha\sin\beta e^{-2i\omega} \end{pmatrix}. \tag{S22}$$

The remaining phase factor $e^{-2i\omega}$ can be compensated using feed-forward operation by a conditional phase gate $W = \exp(-i\sigma_Z\omega)$. With these corrective local phase gates V and W, the coupling operations U_{PSWAP} and (4) are equivalent in the context of the protocol.

6 Characterization automated phase alignment

Here we describe details of the procedure for setting the phase φ in the displaced Sagnac interferometer, which determines the coupling strength of the partial SWAP gate, $\omega = \varphi/2$. First, we block the input B to the interferometer and set all waveplates in the experiment to 0 degrees. We count the single photons at the output ports of the interferometer and scan the piezo voltage to obtain a reference interferogram. Using this reference, we calculate the set point intensity corresponding to the desired phase. We coarsely set the piezo voltage for the target phases from 10 to 170 degrees using the reference interferogram. We then tune the piezo voltage using feedback proportional to the error signal until the observed intensity reaches the set point within a defined tolerance. We resort to scanning the piezo voltage for phases between 0 and 10 degrees and between 170 and 180 degrees. When the intensity reaches the setpoint, we stop the scan.

We have executed the alignment procedure 15 times for the tested desired coupling strengths ω_n and recorded the actual coupling strength ω_e. We have identified failed alignment attempts as cases where the size of the deviation $\Delta = \omega_e - \omega_n$ was greater than 5 degrees. In Fig. S1(a) we compare the desired coupling strength ω_n with the strength ω_e achieved by successful alignment. Their respective deviation Δ is plotted in panel (b). The deviation of ω_e alignments from the nominal ω_n is smaller than 1.1 degrees RMS.

The reliability of our alignment procedure is limited mainly by the hysteresis of a piezo-spring system. Due to hysteresis, the interference fringe generally moves relatively to the previously measured reference fringe. When we execute proportional-feedback loop tuning in flat regions of the fringe near the extremes, we sometimes exceed the allowed voltage on the piezo or exceed the maximal limit of iterations, and the procedure fails. In the main experiment, we have always checked whether the phase alignment succeeded, and in the case of failure, we have repeated the phase alignment.

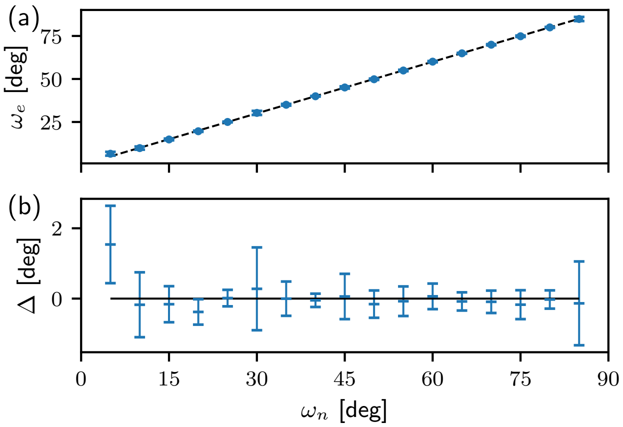

Figure S1. (a) Achieved coupling strength ω_e after successful phase alignment. (b) Coupling strength set deviation of successful alignments. All panels are plotted against the nominal desired coupling strength ω_n. Error bars represent one standard deviation.
